# X-ray diffraction and second harmonic imaging reveal new insights into structural alterations caused by pressure-overload in murine hearts

**DOI:** 10.1038/s41598-020-76163-6

**Published:** 2020-11-09

**Authors:** Jan-David Nicolas, Amara Khan, Andrea Markus, Belal A. Mohamed, Karl Toischer, Frauke Alves, Tim Salditt

**Affiliations:** 1 Institute for X-Ray Physics, Georg-August-Universität Göttingen, Friedrich-Hund-Platz 1, 37077 Göttingen, Germany; 2 Translational Molecular Imaging, Max-Planck-Institute for Experimental Medicine, Hermann-Rein-Straße 3, 37075 Göttingen, Germany; 3 Clinic for Cardiology and Pneumology, University Medical Center Göttingen, Robert-Koch-Straße 40, 37075 Göttingen, Germany; 4 DZHK (German Centre for Cardiovascular Research), Partner Site Göttingen, 37075 Göttingen, Germany; 5 Clinic for Hematology and Medical Oncology, Institute for Diagnostic and Interventional Radiology, University Medical Center Göttingen, Robert-Koch-Straße 40, 37075 Göttingen, Germany; 6 Cluster of Excellence “Multiscale Bioimaging: from Molecular Machines to Networks of Excitable Cells”, University Medical Center Göttingen, Robert-Koch-Straße 40, 37075 Göttingen, Germany

**Keywords:** Structural biology, Biological physics, Nonlinear optics, X-rays, Cardiovascular biology, Cardiomyopathies, Heart failure, Hypertension, Imaging

## Abstract

We demonstrate a label-free imaging approach to study cardiac remodeling of fibrotic and hypertrophic hearts, bridging scales from the whole organ down to the molecular level. To this end, we have used mice subjected to transverse aortic constriction and imaged adjacent cardiac tissue sections by microfocus X-ray diffraction and second harmonic generation (SHG) imaging. In this way, the acto-myosin structure was probed in a spatially resolved manner for entire heart sections. From the recorded diffraction data, spatial maps of diffraction intensity, anisotropy and orientation were obtained, and fully automated analysis depicted the acto-myosin filament spacing and direction. X-ray diffraction presented an overview of entire heart sections and revealed that in regions of severe cardiac remodeling the muscle mass is partly replaced by connective tissue and the acto-myosin lattice spacing is increased at these regions. SHG imaging revealed sub-cellular structure of cardiac tissue and complemented the findings from X-ray diffraction by revealing micro-level distortion of myofibrils, immune cell infiltration at regions of cardiac remodeling and the development of fibrosis down to the scale of a single collagen fibril. Overall, our results show that both X-ray diffraction and SHG imaging can be used for label-free and high-resolution visualization of cardiac remodeling and fibrosis progression at different stages in a cardiac pressure-overload mouse model that cannot be achieved by conventional histology.

## Introduction

Heart failure is a common disease amongst the elderly population entailing a high mortality rate. Both structural and functional changes contribute to heart failure. The deposition of extracellular matrix (ECM) either within the interstitial space or by replacement of the cardiac muscle is a common histopathological finding accompanying heart failure^1–4^ . Progression of fibrosis involves inflammatory stimuli, which in turn initiate pro-fibrotic signaling. ECM remodeling and impaired cardiac muscle function are hallmarks of cardiac fibrosis which consequently lead to increased stiffness of left ventricle and impairment of mechano-electric coupling, thus causing arrhythmias. Cardiac fibrosis can occur due to aging and other heart-related pathophysiology such as hypertensive heart disease^2,5^. Transverse aortic constriction (TAC) is a well-established animal model of pressure-overload induced cardiac hypertrophy and failure. TAC mimics hypertensive heart disease in patients correlating to the clinical manifestation of pressure overload. Consequently, it causes cardiac hypertrophy and interstitial fibrosis in mice with an enlarged ventricular mass^6,7^.

While the importance of cardiac remodeling in heart failure is undisputed, the molecular structural changes involved in the progression of cardiac hypertrophy and fibrosis have so far not been at the focus of medical research. Moreover, there is a lack of understanding of the molecular mechanisms that underlie the interaction of cardiomyocytes with surrounding extracellular components in the healthy and fibrotic myocardium. For this purpose, imaging techniques are required to visualize cardiac remodeling of fibrotic and hypertrophic hearts. So far only histology and immunohistochemistry (IHC) are used as standard techniques for assessing the structural composition of the myocardium but these methods lack adequate resolution and specificity to visualize sub-cellular structures. Ideally, the imaging techniques should be able to probe the molecular structure and alterations thereof associated with pathologies, and at the same time also cover the entire cross section of a heart at sub-molecular level to find and to quantify the affected regions.

Alterations in heart muscle at the molecular level can be studied by using X-ray diffraction. For example, X-ray diffraction can be used to assess kinetics of myosin binding in actively contracting muscles^8–11^. One main disadvantage of the classical muscle diffraction technique is the requirement to extract intact muscle fibers from the heart. Contrarily, recent progress in X-ray optics has made it possible to focus the beam down to a few micrometers and even nanometers^12^. By sacrificing resolution in reciprocal space, millions of X-ray diffraction experiments can be performed sequentially by raster-scanning the X-ray beam over the sample and collecting a diffraction pattern in each spot^13^. Such an experiment offers real-space resolution and structural information, most importantly filament lattice spacing, filament orientation and degree of filament alignment. Spatially-resolved X-ray diffraction has been used for single cardiomyocyte imaging, where it was combined with STED microscopy to obtain a multiscale overview of cardiac sub-micron structures^14^. Most recently, these cells could even be imaged alive in a hydrated environment^15^. The overall tissue structure was so far only imaged in a proof-of-concept study^12^ on a single tissue section of a non-pathological heart and later on a thin section of engineered cardiac tissue^16,17^.

For the present analysis more than 17.8 million diffraction patterns each with a size greater than 4 megapixels were analyzed, which requires a fast detection scheme coupled with a fast and robust data analysis. This is to date the largest screening of cardiac and more generally hydrated soft biological tissue by means of spatially-resolved X-ray diffraction.

A second powerful label-free imaging method for cellular scale visualization of biological tissue is second harmonic generation (SHG) imaging^18^. In contrast to X-ray diffraction it does not depend on or require long-range or medium-range molecular order, but only relies on molecular inversion symmetry. It is based on the non-linear optical effect of SHG in non-centrosymmetric polarizable medium. Accordingly, in a coherent optical process the combined absorption of two excitation photons results in the generation of a single photon of exactly twice the energy and half the wavelength of the original excitation wavelength^19^. Interestingly, both collagen and myosin fibers generate intrinsic SHG, making this imaging approach ideal for investigating ECM remodeling and muscle integrity following fibrosis and hypertrophy in cardiac tissue^20,21^. Despite the remarkable advantages of SHG imaging over conventional histology such as high resolution, specificity and reproducibility, the use of this approach for investigating cardiac remodeling in diseased tissue is still in its infancy. SHG imaging of diseased cardiac tissue has so far focused almost exclusively on the detection of collagen as an indicator of ECM remodeling but has not studied the myosin fibers and overall tissue architecture^22^. In fact, studies targeting the pathological alteration in myosin filaments have been performed at cellular level only^23^.

In this study, our primary aim was to establish spatially-resolved X-ray diffraction and SHG imaging for assessing structural changes in fibrotic and hypertrophic hearts obtained from wild-type mice subjected to TAC. Using X-ray diffraction, we demonstrated that the acto-myosin lattice spacing in the areas of sever pathological remodeling of TAC hearts is higher compared to controls and more heterogeneously distributed, and that the semi-crystalline order in damaged regions is slightly reduced. The evaluation of lattice spacing could also reveal structural features, such as a ring-type structure in the myocardium, that would not be observable in classical X-ray diffraction. These finding were further complemented by SHG imaging which revealed sub-cellular deterioration of myofibrils and surrounding tissue due to underlying ECM remodeling and collagen deposition.

## Results

### Comparative imaging of cardiac tissue using X-ray diffraction, SHG imaging, and histology

**Figure 1:**
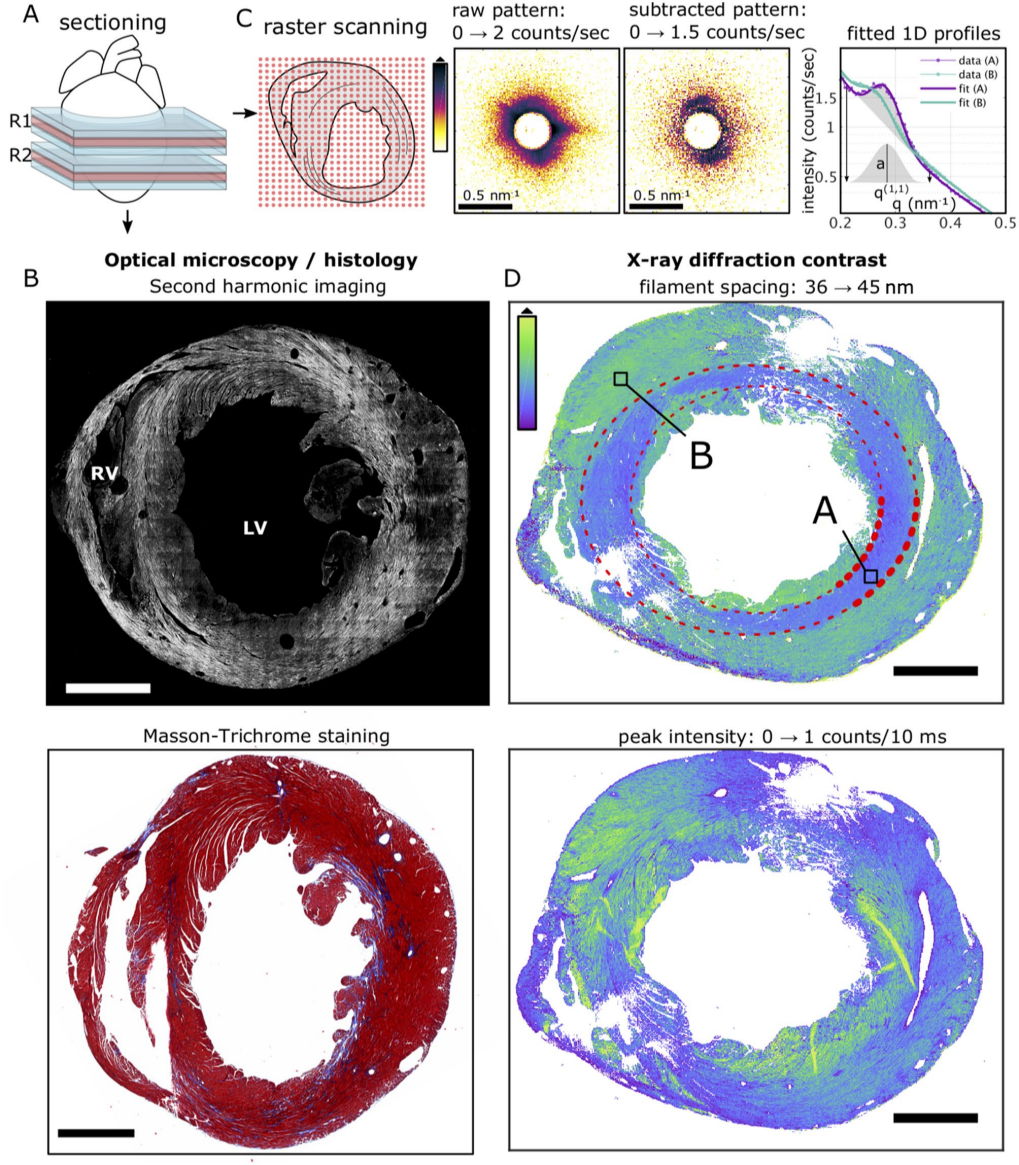
Comparative multi-imaging approach using SHG imaging, histological analysis and X-ray diffraction. **(A)** Hearts were sectioned in a pre-defined order. Tissue sections for X-ray diffraction and SHG imaging (in red) were cut at approximately 1/3 and 2/3 distance from base to apex, namely region 1 (R1) and region 2 (R2), respectively, while 1 mm neighboring blocks (in blue) were used for histology and IHC. **(B)** SHG (top) from myosin and collagen are shown in white and MT stained neighboring tissue section (bottom) depicts hypertrophy and interstitial fibrosis in which muscle is stained red and collagen deposition in blue. **(C)** Data taken by X-ray diffraction were first background-subtracted, angularly averaged and fitted by a model function before a map of a fit parameter such as filament spacing, and peak intensity could be created. **(D)** Filament spacing in the bulk tissue (top) is around 42 nm except for an inner ring marked between the red dotted lines, where 39 nm were measured. Peak intensity (bottom) is highest around the ventricle. All images were recorded on adjacent tissue sections obtained from the same TAC heart at region 2. Scale bar: 1 mm. *RV* right ventricle, *LV* left ventricle.

Mice subjected to TAC-operation developed cardiac hypertrophy within 16 weeks post TAC surgery depicted by significantly higher weights of the TAC hearts (350 mg ± 25 mg; p-value = 0.002) as compared to healthy hearts (150 mg ± 25 mg) (Supp. Section 1, Table S1). Heart failure with impairment of cardiac function was also evident by echocardiography (Supp. Section 1, Table S2). TAC hearts presented different severity of cardiac remodeling and were composed of enlarged cardiomyocytes with an overall increase in ECM content (Fig. S1 and Fig. S2). To perform X-ray diffraction, SHG imaging, histology and IHC on adjacent sections, excised hearts were sectioned according to the protocol sketched in Fig. 1A. Note that the sectioning scheme was adapted to obtain tissue sections from similar regions from TAC and sham hearts. Tissue sections of 30 µm for X-ray diffraction and SHG imaging (in red) were cut at approximately one-third and two-third distance from base to the apex (denoted as region 1 and region 2, respectively), while 1 mm neighboring blocks (in blue) were used for histology. Figure 1 exemplifies the combined use of the complete set of available modalities depicting the analysis of neighboring transverse tissue cross sections obtained from the same TAC heart (TAC 2, see Supp. Section 4–5). The differences in the overall shape of the neighboring heart tissue sections occurred due to serial cutting of tissue sections of varying thicknesses from whole heart and subsequent differences in the mounting of tissue sections for each imaging modality. The paraffin embedding procedure furthermore causes a slight shrinkage of the tissue, which may influence the appearance of the histological slice.

For SHG imaging, two-dimensional (2D) micrographs of 30 µm cardiac tissue slices were acquired using an average power of 40 to 50 mW at a wavelength of 810 nm for SHG emission (Fig. S7). A stitched image of a tissue section is shown in Fig. 1B (upper panel) where the SH emission from the two cardiac harmonophores, i.e. myosin and collagen, are represented in white. Note that the stitched image presents an overview of the SH emission from an entire cardiac section, at a scale where SHG derived from myosin cannot be distinguished from the collagen originated SHG. The histological image (Fig. 1B, lower panel) represents a stitched image of a Masson’s Trichrome (MTS)-stained adjacent heart tissue section from the same heart sample and shows the muscle stained in red while collagen in blue highlights regions of interstitial fibrosis.

X-ray diffraction data acquisition required three pre-processing steps, illustrated in Fig. 1C. The acto-myosin scattering signal was superimposed by intense scattering from the 1 mm thin lead beamstop wire. To perform accurate background subtraction, 20 empty frames in each line were averaged and used to subtract from the raw data in the respective line. The subtracted pattern now clearly shows the (1,1) reflection from the acto-myosin lattice while the (1,0) reflection was absorbed by the beamstop. Subtracted data were then angularly averaged to obtain one-dimensional (1D) profiles of the scattering signal as a function of the scattering angle θ, encoded in the wavevector transfer q = 4π/λ sin(θ/2). The resulting intensity profiles I(q) were then fitted using a model function comprised of a power-law decay and a Gaussian,

$${\text{I}}\left( {\text{q}} \right) = {\text{ S q}}^{{ - {\text{d}}}} + {\text{ I}}_{{\text{p}}} {\text{exp}}\left( { - \left( {{\text{q}} - {\text{q}}_{0} } \right)^{2}/\sigma^{2}} \right) + {\text{ I}}_{{{\text{bgr}}}} ,$$

where the width σ of the Gaussian were fixed to 0.034 nm^−1^, while the parameter S, the decay exponent d, the uniform background intensity I_bgr_ as well as the intensity I_p_ and lateral position q_0_ of the Gaussian component, referred to here as peak intensity and peak position, were obtained from non-linear least square fitting. The lateral peak position q_0_ was then converted to the filament spacing a = 4π/q_0_ of the acto-myosin lattice. All processing steps were performed in MATLAB (MathWorks) using the nano-diffraction toolbox ^12^. From the fitted profiles it is already evident that the filament spacing can vary significantly. Figure 1D indicates that data was averaged within regions A and B.

Figure 1D shows how the filament spacing and the peak intensity, both structural parameters which can uniquely be assessed by scanning X-ray diffraction, vary throughout the cardiac tissue of the TAC heart. In this example, the filament spacing map reveals a central ring (highlighted in red) with lower filament spacing within the bulk of the tissue as compared to the remaining tissue. Three samples were found that exhibited this particular structural feature. The lattice spacing difference between the outer and inner ring was 1.7 nm, 2.2 nm and 2.4 nm, as detailed in Supp. Section 2.

### X-ray diffraction imaging of sham heart tissue

**Figure 2:**
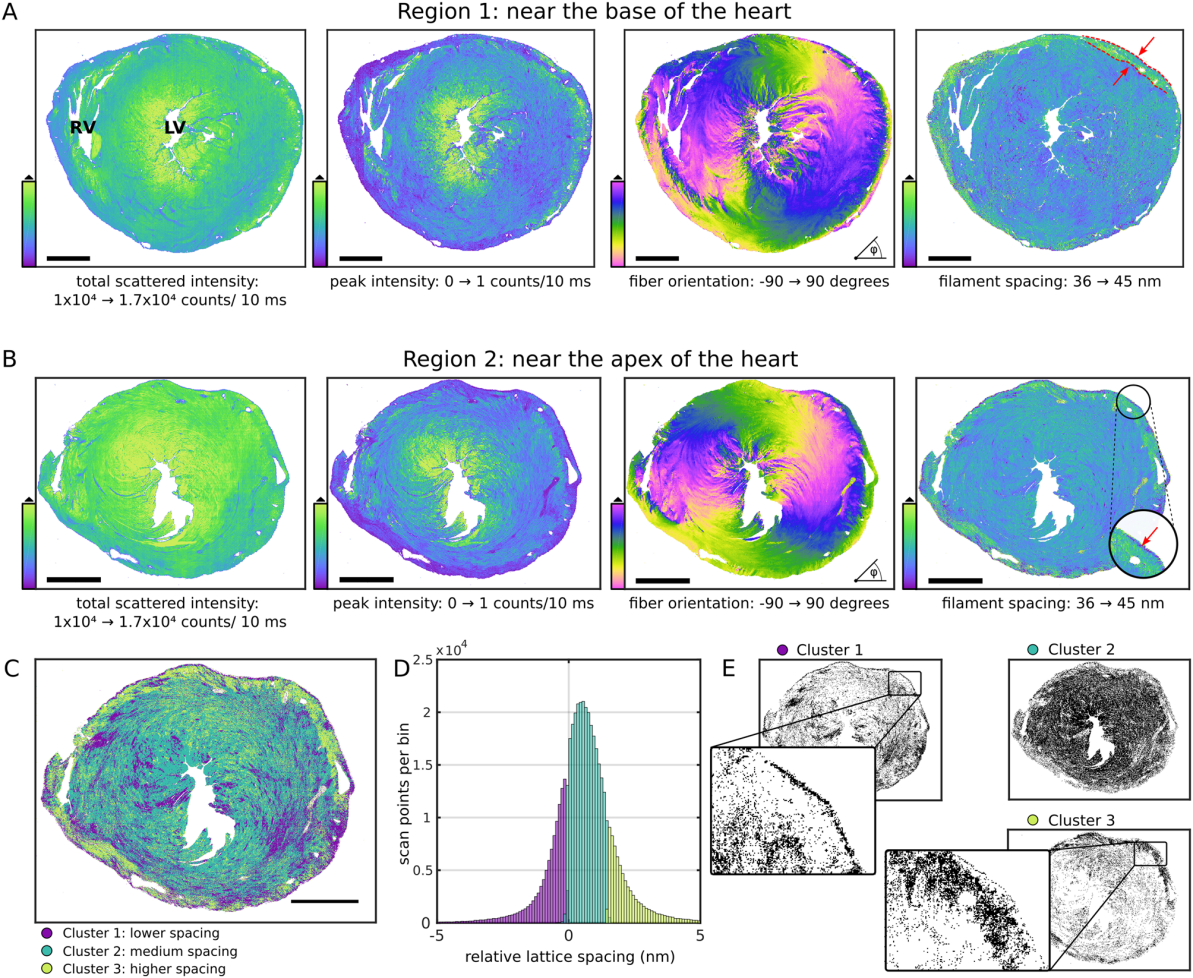
Comparison of structural parameters in region 1 (near the base of the heart) and region 2 (near to the apex of the heart) of a sham heart. **(A)** In region 1, the filament spacing is homogeneous, with the sole exception of a small layer in the outer myocardium where the filament spacing is increased, indicated by red arrows. **(B)** The filament spacing in region 2 resembles the distribution in region 1. In contrast to **(A)**, the thin epicardial layer, indicated by a red arrow in a zoom inset, is now clearly visible. Note that in this layer the filament spacing is reduced. Sections from both regions appear very similar in shape and size. Total scattered intensity and peak intensity is increased around the left ventricle and filaments circulate smoothly around the left ventricle. **(C)** K-means clustering based on the five-dimensional dataset of fit parameters was used to identify three distinct clusters which are color-coded in **(C)**. **(D)** Histograms of lattice spacings corresponding to the three clusters identified in **(C)**. **(E)** Logical maps of the three clusters demonstrate more clearly, that cluster 1 with lattice spacings below 38.9 nm accounts well for the thin epicardial layer while cluster 2 represents the myocardium, with the exception of cluster 3 that highlights the outer myocardial layer with lattice spacings above 40.4 nm. Scale bar: 1 mm. *RV* right ventricle; *LV* left ventricle.

Healthy tissue sections obtained from sham-operated hearts served as a control group. Figure 2 shows one example of two sections taken at region 1 and 2. In both regions, the peak intensity was, with more than 1 counts/10 ms, significantly higher around the left ventricle than the bulk tissue with 0.22 counts/10 ms, estimated from the mode of the intensity distribution. The mode of the filament spacing distribution was determined at 38.6 nm and 38.9 nm in region 1 and 2, respectively, which is consistent with a previous observation by Nicolas et al*.* 2017^12^, where the mode was found at 38.9 nm.

The outer layer of the myocardium, highlighted in red in Fig. 2A, has a higher filament spacing. This can be explained by the fact that these muscle fibers only support the structure of the heart while the inner myocardium generates the force of contraction. The lattice spacing is reduced at the outermost epicardial layer which is about 4–5 µm thick. A reduced lattice spacing, as highlighted in red in Fig. 2B, reflects a stiffer matrix, which is primarily composed of connective tissue. In this region, the peak intensity is well below the mode of the distribution (Fig. 2B). It is even discernible that the epicardial layer in region 2 was slightly more prominent. This follows the intuition that region 2, i.e. the region closer to the apex of the heart, more strongly generates the force of contraction to pump blood into systemic circulation.

In fact, a segmentation of the data obtained in region 2 based on k-means clustering with three clusters could be used to identify the outermost epicardial layer with a lower lattice spacing and the outer myocardial layer with a higher lattice spacing, respectively. For discrimination of the three clusters, all five fit parameters, namely *S, d, I*_*p*_*, q*_*0*_ and* I*_*bgr*_, were used. The map of clusters is shown in Fig. 2C together with a histogram of lattice spacings (Fig. 2D) that resulted from the clusters. Maps of all scan points belonging to one of the three clusters are shown in Fig. 2E. The outermost epicardial layer can be clearly seen in cluster 1, relating to a cluster with lower lattice spacings (below 38.9 nm). Lattice spacing corresponding to cluster 1 is also heterogeneously distributed within the myocardium which provides additional information about network of tightly packed tissue that might play a role in maintaining the overall structural integrity of the heart. The bulk of the tissue is composed of lattice spacings between 38.9 nm and 40.4 nm. The third cluster clearly highlights the outer myocardial layer with lattice spacings above 40.4 nm. The arrangement of the layers of the cardiac wall relative to filament spacing can provide additional cues about the mechanisms governing the cardiac contractility in healthy and diseased hearts.

### X-ray diffraction, SHG imaging and histology of sham and TAC heart

Based on the 2D scattering patterns, the total scattered intensity and cardiomyocyte chain orientation or fiber orientation can be inferred. To calculate the former, it is sufficient to integrate the scattered intensity, while determining the cardiomyocyte chain orientation requires radial averaging of the 2D patterns within 0.2 – 0.5 nm^-1^, yielding the 1D azimuthal intensity profile I(φ). I(φ) was normalized

$$I_{norm} (\varphi ) = \frac{I(\varphi ) - \min (I(\varphi ))}{{\sum\limits_{\varphi } {I(\varphi )} }}$$

and the circular mean and circular variance (Var) were calculated. The circular mean angle φ_0_ of the scattering is rotated 90º with respect to the cardiomyocyte chain orientation. Anisotropy can now be defined as 1 − Var(I_norm_(φ)), yielding values ranging between 0 and 1. Zero anisotropy resembles no orientational order, while an anisotropy of 1, i.e. zero variance, would be indicating perfect orientational order.

**Figure 3:**
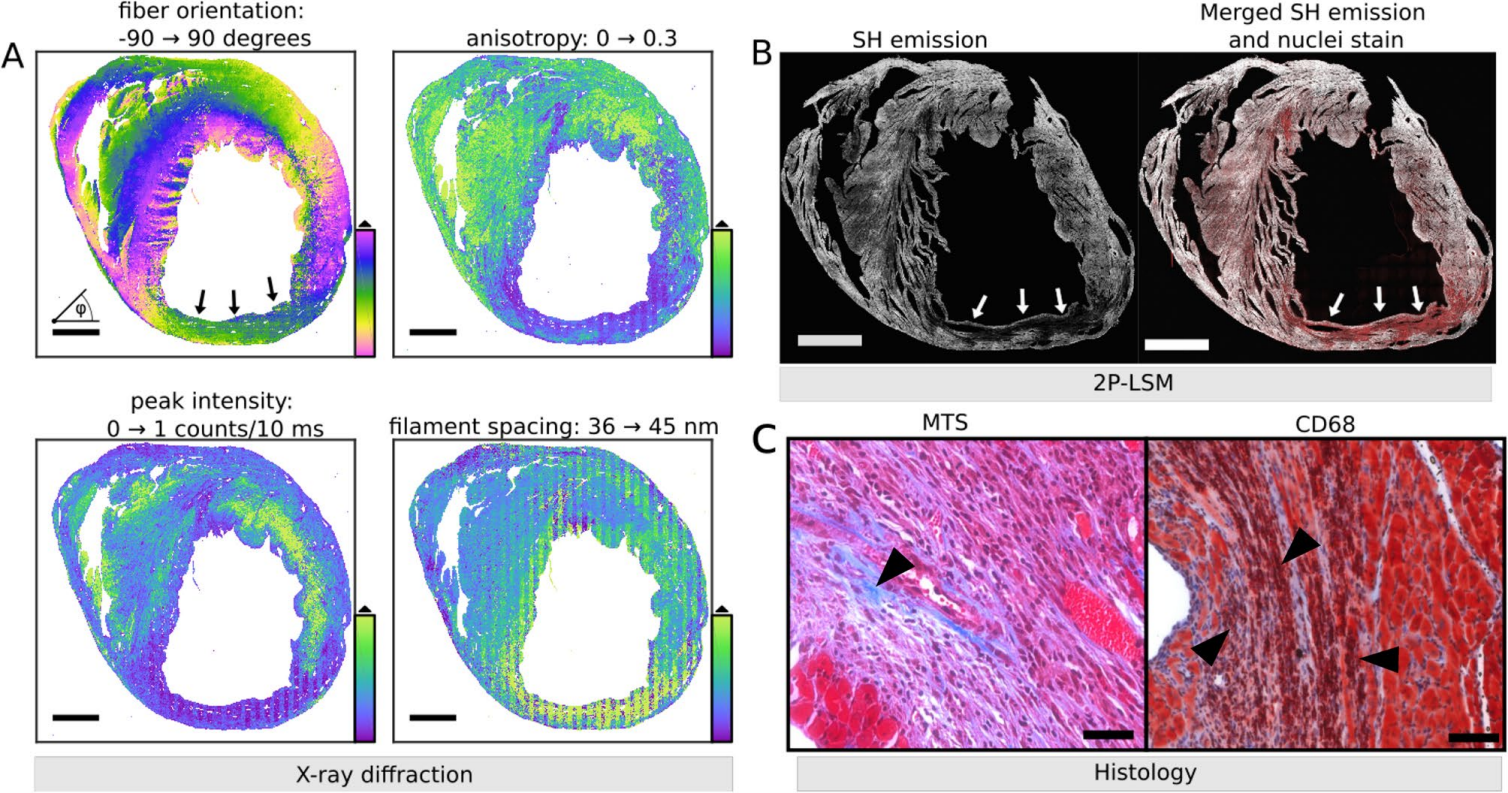
Images of tissue sections from the same TAC-operated heart with a region of severe pathological remodeling (pointed by arrows). **(A)** X-ray diffraction of this region (marked by black arrows) is distinguished by a lower anisotropy, a peak intensity close to zero and an increased filament spacing surrounding the affected tissue area while the primary orientation of the tissue components is maintained. For this reason, the fit diverges and the filament spacing reaches the fit boundary value of 45 nm. **(B)** SHG imaging shows negligible SH emission from myosin and collagen at this region and a higher signal from the DRAQ5 nuclear stain in red (marked by white arrows) which indicates a high cell infiltration. **(C)** Magnified histological images of this region reveal a limited deposition of collagen in blue as shown by the MTS (left image, marked by black arrowheads) and a high infiltration of positively stained macrophages shown by anti-CD68 IHC (right image, marked by black arrowheads). Scale bar in **(A,B)** 1 mm, scale bar in **(C)** 50 µm. *RV* right ventricle; *LV* left ventricle.

In Fig. 3A, filament orientation, anisotropy, peak intensity and filament spacing are shown for one of the TAC hearts that represented severe cardiac remodeling at a localized region serving as an interesting example to present a comparison between X-ray diffraction and SHG imaging. The spatial maps from X-ray diffraction, reveal that a low anisotropy and peak intensity coincides with a larger filament spacing. In this example, the localized region of cardiac remodeling at R2 of the TAC heart can be found by thresholding the lattice spacing map at 46.0 nm, as shown in Supp. Section 3. The area is then characterized by an anisotropy of 0.12 and an intensity of 0.11 counts/s, i.e. most myocardium has been replaced by fibrotic, non-muscle tissue indicating replacement fibrosis at this region. In contrast, the entire sample is characterized by an anisotropy around 0.51 and an intensity of 0.27 counts/10 ms. This shows that, without the presence of intense reflections, there is only residual alignment and anisotropy reflects the orientational order of all scattering components of the tissue.

The distribution of filament spacings together with maps of structural parameters for all 12 imaged sections are shown in Supp. Section 4. The average mode of all sham heart sections was 39.0 ± 0.3 nm and the lattice spacings in Figure S3 and Figure S6 are shown relative to this reference value to highlight any relative changes. The distribution of R2 of sample TAC 3, representative for a sample with a central ring, was bimodal with two modes at 38.7 nm and 40.3 nm.

Corresponding to these findings, Fig. 3B shows that the SH emission from both myosin and collagen is only sparsely present or even completely absent in this severely affected localized region of the TAC heart adding further evidence to the finding that this region had replacement fibrosis (Supp. Section 5, Fig. S7). Further, in this TAC heart the presence of high cell infiltration was also observed by DRAQ5 nuclear staining. An overall 78% increase in cell infiltration (Supp. Section 6, Fig. S8) in TAC 3 heart indicates an active immune response which was confirmed by positive immunostaining of immune cells using antibodies against the leucocyte marker CD45 (Fig. S8A-B) and the macrophage marker CD68 (Fig. 3C and Fig. S8C-D). Furthermore, IHC also showed a 65% increase in the expression of alpha-smooth muscle actin within the myocardium of this TAC heart as compared to sham hearts, indicating the presence of active myofibroblasts (Fig. S8E-F).

### SHG imaging of sham heart tissue

**Figure 4:**
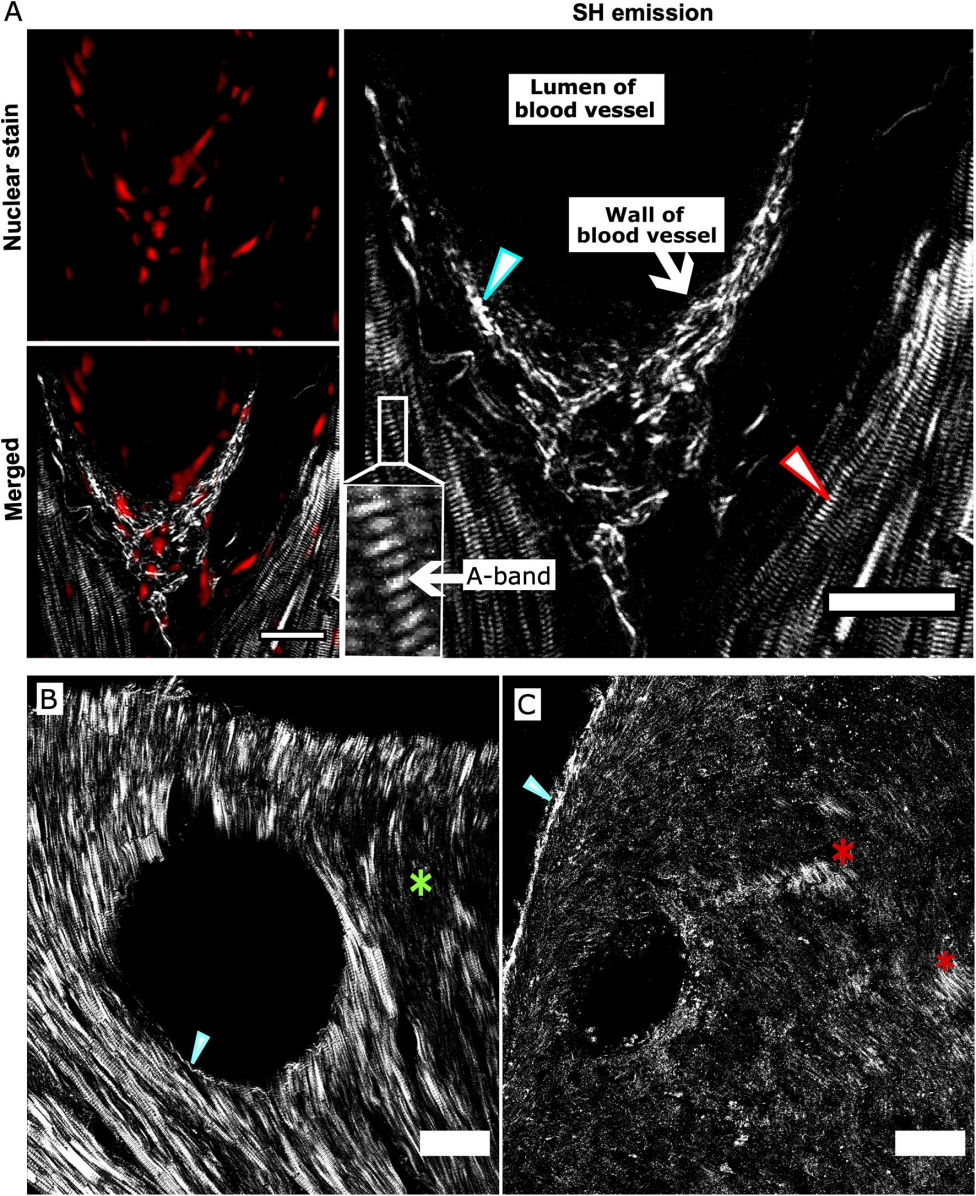
Representative 2D images of SH emission from myosin and collagen in a sham heart. **(A)** A region around a coronary blood vessel is shown including the images for DRAQ5 nuclear staining of cells, the merged image and an enlarged image for SH emission for clear visualization. The blue arrowhead marks the SH emission from collagen fibers localized around the blood vessel and the red arrowhead shows the SHG originating from myosin emitting a typical striated pattern. The Anisotropic band (A-band) is marked in the zoomed-in image of the myofibril. **(B)** SH emission from myosin fibers lying in the imaging plane except for the region marked with a green star. **(C)** SHG from myosin fibers which are not aligned in the imaging plane except for regions marked with red stars. Collagen-emitted SHG is marked by blue arrowheads which is present in **(B)** around the blood vessel and in **(C)** at the epicardium. Scale bar: 20 µm **(A)** and 50 µm **(B,C)**.

SHG imaging was performed to gain detailed information on the structural disruption of the cardiac tissue at sub-cellular level. A 2D image of a coronary blood vessel of a sham heart with representative images for DRAQ5 stained nuclei, SHG and the merged signal is shown in Fig. 4A. SH emission from myosin and collagen can be clearly distinguished as they present distinct structural morphology. The SHG from collagen fibers shows fibrous structures, which in healthy hearts were only visualized at localized regions such as around the large coronary blood vessels (Fig. 4B). Myofibrils are the main components of the cardiac muscle mass and hence myosin SHG signals were abundantly present throughout the heart. Within the sarcomere, the SHG signal originates from the A-band, which is the region primarily composed of thick myosin filaments. The A-band is separated by an isotropic region (I-band) composed of actin filaments which appear dark as they do not emit SH signal, resulting in the striated pattern of the myofibril (Fig. 4A). However, the efficiency of a myosin-specific SH signal was found to be strongly dependent on various factors including the orientation of the myofibrils, the focus of the imaging plane and the physical and virtual cut of the tissue. If the filament axis was lying in the imaging plane the characteristic striated pattern was resolved and had a high SHG signal intensity (Fig. 4B), which was weaker otherwise (Fig. 4C). On the other hand, the intensity of collagen specific SHG was less depended on the orientation but was mainly affected by the thickness and quantity of collagen fibers (Fig. 4B,C).

### Collagen-emitted SHG in sham and TAC heart tissue

**Figure 5:**
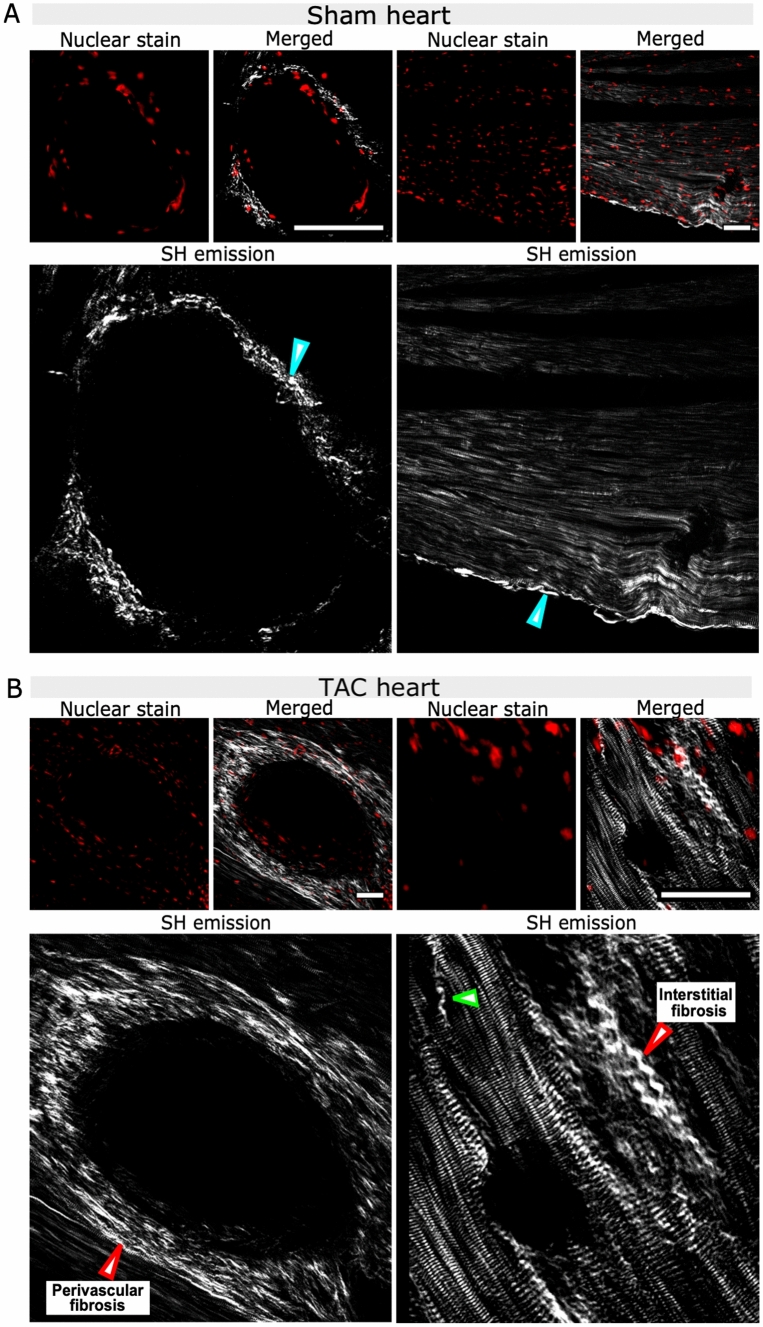
Representative images of the regions with collagen specific SHG in sham and TAC-operated hearts including DRAQ5 nuclear staining for visualization of cells, merged images for nuclear stain and SHG signal and enlarged images for SH emission for clear visualization. **(A)** Collagen-specific SHG signals in the sham heart were present around the coronary blood vessels and the epicardium (blue arrowheads). **(B)** SHG in the TAC hearts revealed the development of perivascular and interstitial fibrosis (red arrowheads). Individual collagen fibers were also visualized within the muscle mass in TAC hearts (green arrowhead). Scale bars: 50 µm.

Furthermore, the comparison of images of whole tissue sections from sham and TAC-operated hearts revealed the difference in the overall morphology and distribution of collagen and myosin represented by their SHG signal (Fig. 5). In sham hearts the collagen fibers were mainly visualized around the large coronary vessels and the epicardium (Fig. 5A). In comparison, the collagen emitted SHG intensity in TAC hearts was significantly higher (p-value = 0.015) (Supp. Section 7, Fig. S9A and C) around the coronary vessels signifying the development of perivascular fibrosis. This was further validated by histology which showed similar results (p-value = 0.039) (Supp. Section 7, Fig. S9B and D). SHG also revealed the presence of collagen fibers within the interstitial space at sub-micron scale in TAC hearts, allowing the visualization of the individual collagen fibrils surrounded by myofibers (Fig. 5B).

### Myofibril morphology in sham and TAC hearts

**Figure 6:**
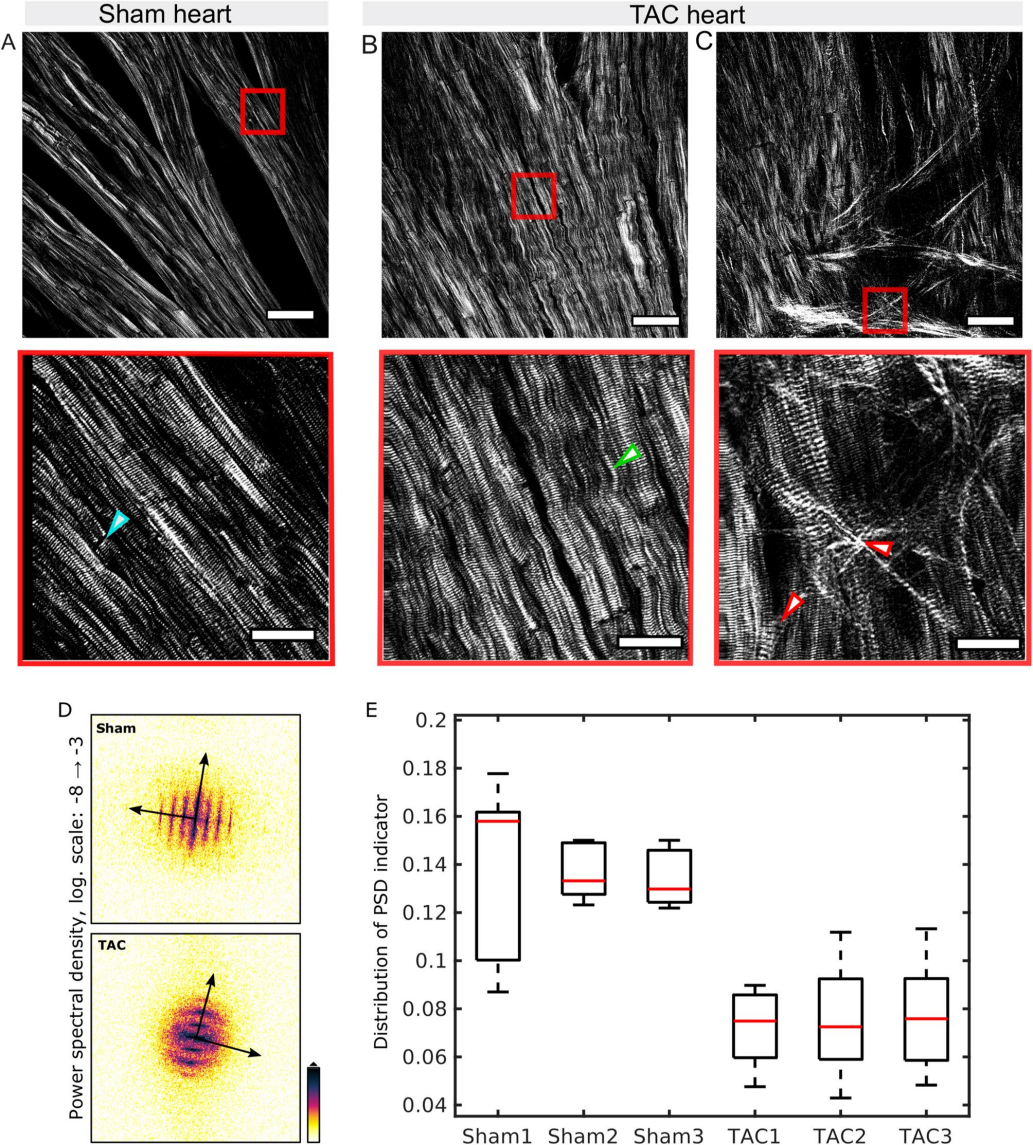
Representative images of SHG showing the arrangement of myosin fibers in sham and TAC-operated hearts with overview images (top) and magnified images at the ROI (bottom). **(A)** The myosin fibers show a linear and symmetric arrangement in the sham heart. The intercalated disks between myosin fibers do not emit SHG and appear dark which is pointed by a blue arrowhead. **(B)** In the TAC heart, undulated myosin fibers are located within the muscle mass as shown by a green arrowhead. **(C)** The cross-linked collagen fibers can be seen in the myocardium causing disruption and rupture of myosin fibers indicated by red arrowheads. Scale bars: 50 µm in overview images (top panel) and 20 µm in magnified ROI images (bottom panel). **(D)** Image analysis of myosin morphology in SHG micrographs of TAC and sham hearts. The power spectral density (PSD) was computed for each image, see the example of a TAC (bottom) and sham micrograph (top) by 2D fast Fourier transformation (FFT). The 2D PSD shows a pattern reflecting the orientation of myosin strands, the periodicity of the striation as well as the degree of undulation. **(E)** After azimuthal average, a single scalar indicator value can be computed from the 1D PSD, which differs between TAC and sham morphology with high statistical significance (see main text).

SHG from myosin in sham heart tissue showed a linear arrangement of myofibrils. The intercalated disks between individual muscle fibers appear as dark regions owing to the fact that these areas do not generate SH signals (Fig. 6A). By contrast, areas with severe pathological tissue remodeling were found in TAC hearts, which contained distorted myosin fibers. These regions were comprised of abnormal and undulated myosin fibers that lacked periodicity and symmetry, features which were present in healthy hearts. Interestingly, the collagen fibers in these areas were sparsely present or completely absent (Fig. 6B). Such regions of abnormal myosin filaments were abundant at the wall of the left ventricle and within region 2, i.e. near the apex of TAC hearts.

SHG imaging also revealed that the deposition of collagen fibers has detrimental effects on myosin fiber morphology. An excess of collagen seemed to have caused the displacement of myofibrils which might also contribute to ruptures and changes in the arrangement of the surrounding myofibrils (Fig. 6C).

Next, we investigated whether the morphological changes in the myosin fibers described above for exemplary images, can be quantified by an automated image processing scheme and whether the group differences in morphology between TAC and sham stand up against statistical tests. To this end we designed a Fourier-based image processing workflow, starting with a 2D FFT operation for each micrograph. The resulting power spectral density (PSD) given by the squared modulus of the Fourier image was plotted which reflected the orientation of myofibril strand, the striation periodicity in terms of the corresponding lattice peaks, as well as the degree of undulation (Fig. 6D). The 2D PSD graphs were then analyzed with respect to their two main axes of symmetry determined by a principle component analysis (PCA)^24^. Since the undulation was identified as the main characteristic feature of TAC morphology, a PSD-based indicator metric was computed as follows. Starting from the center of the PSD image, a cake integration was performed covering the two principal axes. The opening angle of this integration cone (‘cake’) was then varied between 5° and 20°. Division of a 1D PSD curve computed with larger angle by a curve computed with lower angle then yields an indicator curve. The larger the amount of (partial) rotational averaging by the undulations, the smaller the deviation of the indicator curve from a value of 1. This was finally captured by a simple root-mean-square analysis yielding a single scalar value for the PSD-indicator, as displayed in the box-whisker plot of (Fig. 6E). This was achieved by comparing a total of 20 TAC micrographs and 20 sham micrographs from 3 TAC and 3 sham hearts, respectively (Supp. Section 8). The distributions of the PSD-indicator for 3 TAC and 3 sham intra-group averages were then subjected to a two-tailed paired Student’s t-test, indicating a p-value of p = 0.0011. In summary, these findings show that myofibrils in TAC hearts are irregular and distorted as compared to the very linear arrangement in sham hearts.

## Discussion

In this proof of concept study, we demonstrated that spatially-resolved X-ray diffraction and SHG imaging enables an additive and comprehensive assessment of myosin filaments, revealing structural disruption of the myocardium due to fibrosis and hypertrophy in a mouse model of cardiac pressure-overload.

X-ray diffraction reveals the cardiomyocyte chain orientation throughout the whole myocardium (fiber orientation), the degree of chain alignment or anisotropy, the peak intensity reflecting the predominance and ordering of the acto-myosin structure, as well as the filament spacing as the most important structural parameter of the acto-myosin assembly. Together, the spatial maps of these parameters unveil the cardiac micro-architecture underlying its mechanical function. Importantly, this study shows how the scale-bridging ability of spatially-resolved X-ray diffraction arises from its intrinsic combination of information from real- and reciprocal space, enabled by automated analysis of millions of diffraction patterns. Compared to control (sham) hearts the diffraction signals in TAC hearts reveal structural abnormalities in the myocardium which exemplifies the ability of X-ray diffraction to elucidate pathological structure alterations. Based on 2D maps of filament spacing and peak intensity, we could identify fibrotic lesions and observe an increase in lattice spacing in the surrounding tissue. In sham hearts, only the outer myocardial layer coincides with an increased lattice spacing. This points to the fact that regions with a higher amount of connective tissue may be associated with a different structure of the acto-myosin assembly. Since at this point, we lack a model according to which the lattice spacing of thick and thin filaments can be put into direct relation to mechanical properties, the increased values are difficult to interpret, but may speculatively be attributed to a softer muscle structure. In this simplified picture, the outer myocardium, which is also characterized by an increased lattice spacing could potentially also accommodate a relaxed filament packing, in contrast to the inner myocardium which generates the contractile force.

Fibrotic tissue was found to also yield a low anisotropy, i.e. orientational order. This indicates that the replacing tissue components, although maintaining the original orientation of the tissue, do only pertain a residual degree of alignment. Mapping the filament spacing can also reveal ‘hidden features’ of the cardiac architecture. For example, we have found very distinct regions in the bulk of the tissue where the filament spacing was significantly lower than in the remaining tissue. We therefore hypothesize that sheets of myocardium that are required to generate a larger force are composed of muscle fibers with denser filament packing. To this end, correlating the diffraction results to a full 3D vector field mapping of the cardiomyocyte chain orientation, as it can be obtained from X-ray tomography, could potentially show whether such sheets can be delineated and distinguished both in their radial and helical angles as well as acto-myosin structure.

All TAC hearts presented different degrees of severity of cardiac remodeling which were successfully identified by using both techniques. For instance, one of the TAC hearts presented an active immune response and replacement fibrosis at the left ventricular wall. The regions of high cell infiltration in this TAC heart lacked the acto-myosin diffraction signal, and also the SHG emission from myosin and collagen fibers, which signifies the presence of activated myofibroblasts and the transition of healthy cardiac tissue to a fibrotic subtype^20,21^. By contrast, the two other TAC hearts did not show an active immune response but presented severe hypertrophy in the presence of either interstitial and/or perivascular fibrosis. Thus, the combined use of X-ray diffraction and SHG imaging corroborated the finding that two stages of fibrosis were observed: an active onset of fibrosis with immune response and an end-stage fibrosis with collagen deposition and ECM turnover accompanied by myofibril distortion^3,5,25,26^.

Notwithstanding the unique capability of scanning X-ray diffraction, we must also briefly discuss the detrimental effect of radiation damage. As discussed in Nicolas et al. 2019^16^, the acto-myosin reflection is sensitive to dose and dose rate, controlled by the various scan parameters. For this reason, we here chose a fixed exposure time of 10 ms, step sizes around 5 μm and a spacing of 5 μm between adjacent scan lines to avoid illumination of areas of the tissue which have already received a significant dose before and therefore do not reflect the undamaged structure. While the applied dose of 2.54 MGy still exceeds the tolerable dose of 0.5 MGy we can assume that by this ‘fresh spot’ illumination strategy, the sample is largely unaffected by radiation damage^16^. In particular, given this particularly careful choice of raster-scanning parameters, the structural parameters such as filament spacing are not affected as demonstrated in Nicolas et al. 2019^16^. The overall scan time required for one scan was, on average, approximately 8 h. This time will be significantly reduced in future experiments, given the brightness increase resulting from current and future synchrotron upgrades.

Complementing and extending these findings, features attributed to fibrosis and hypertrophy, starting from single collagen fibrils to deteriorated myofibrils, were also visualized by SHG imaging. In particular our results demonstrate the ability of SHG imaging to visualize cardiac structure at sub-cellular scale which can be used to detect and distinguish SHG from the myosin and collagen fibers. Tiaho et al*.* 2007 showed that SHG from myosin and collagen can be distinguished in gastrocnemius muscle when excited using the same wavelength^16,27^. Our results add to this finding by presenting SHG imaging in healthy hearts in comparison with hypertrophic and fibrotic hearts highlighting the possibility to visualize structural discrepancies in pathological heart tissue. This approach could be further exploited in future to study the detrimental effects of other pathologies on the most relevant structural and functional constituents of the heart, i.e. collagen and myosin^28–31^. Previous work has further shown that changes in the SHG signal intensity can yield structural information on a crystallographic level, such as the semi-crystalline order of the A-band^32^ or the helical myosin filament angle^16^. Here, by scanning X-ray diffraction, we can now correlate a spatially varying SHG intensity to the changes in the crystallographic filament packing of acto-myosin.

We have also shown that morphological changes attributed to heart failure can be successfully identified at sub-micron scale using SHG imaging including reliable detection of interstitial and perivascular fibrosis and myofibril distortion at tissue level. Yuan et al*.* 2019 investigated the structure of cardiac myosin filaments in a pressure overload mouse model^33^. They reported changes in the myosin structure in a cell stretch model but found no differences in morphology of the myocardium at tissue level. By contrast, we observed pronounced differences in the sub-cellular architecture of the myocardium in healthy and diseased hearts including collagen deposition and disruption of myosin fibrils at high-resolution (Fig. 6). The discrepancy in these findings are attributed to the fundamental differences in the methodology including the imaging approach. For instance, in the previous study polarized SHG imaging was performed to image cryofrozen tissue sections in relatively small fields of view (FOV)^33^. While we performed non-polarized SHG imaging on fixed 30 µm vibratome tissue sections obtained from different regions of the hearts which were not frozen. Further, we also acquired overview scans for probing entire tissue sections and then performed subsequent high-resolution imaging at an ROI. Most importantly, in our study SHG imaging was performed on relatively severely affected hearts that were obtained 16 weeks post TAC operation, while in the previous study hearts were imaged 4 weeks after TAC which had early onset of cardiac remodeling only at single-cell level^33^.

The observed disruption of myofibrils that may occur due to the cross-linking of collagen fibers as a result of ECM remodelling has not been reported before. This might be due to the fact that histology is the most commonly used conventional diagnostic tool for accessing cardiac pathology which is not able to visualize the micro-abrasions and cannot resolve composition and interaction of individual myofibrils and collagen fibers within the heart tissue. Moreover, unlike histology, SHG imaging is a label-free imaging approach which has high specificity and hence avoids complications like unspecific binding and overstaining of tissue slices and therefore saves a significant amount of time that is usually needed for the staining process.

In healthy cardiac tissue the myofibrils exhibited a regular and periodic organization while in the diseased hearts the myofibrils had irregularities in the periodicity. In certain myopathies inclusion of non-sarcomeric structures can cause damage to microarchitecture of the muscle^34^. In TAC mouse model, the pressure-overload induced hypertrophy and fibrosis may have resulted in undulated and twisted and/or ruptured myofibrils. Since this phenomenon was not observed in sham hearts, it was not an artefact from sample preparation. We postulate that in fibrotic hearts, underlying ECM remodeling and collagen deposition in surrounding regions may cause undulation by displacing the myosin filaments within the cardiac muscle mass. However, further testing would be necessary to confirm this claim by analyzing thicker tissue samples in 3D obtained at different time points after TAC operation and also by looking into different cardiac pathologies. Since the sarcomere is the fundamental element of cardiac contractility, the reported micro-modulations of myofibrils can also help explain the contractile dysfunction and arrythmias known to occur due to cardiac failure.

## Conclusion and future prospective

Combined results from the two imaging modalities contribute to the understanding of the pathology of cardiac pressure-overload by providing unprecedented visualisations of pathological alterations in heart tissue samples. These findings are beyond the scope of conventional histology and IHC which cannot provide information on the crystallographic structure of acto-myosin filament lattice. Further, histology also lacks the resolution and specificity of SH intrinsic signal. Importantly, the recorded signals lend themselves to ample quantification, which in future can be extended well beyond the current level. The present study already provides a blueprint for such future studies, by comparing the structure of healthy and pathogenic hearts. The characteristic alterations of the micro-architecture in the diseased heart such as disarrayed myocardium, deterioration and rupture of myofibrils and interaction of ECM with surrounding tissue may possibly also serve as “imaging biomarkers” in future diagnostic applications. Given prior combined and mutual corroboration of synchrotron X-ray and SHG imaging results for validation studies performed on cardiac biopsies, one would ultimately be able to base a diagnostic decision only on SHG imaging alone, since it is the clinically much more accessible technique^35,36^. Finally, we hope that by revealing pathological alterations during different stages of cardiac remodelling, advanced imaging of cardiac structures using SHG as compared to histology will provide precise and reliable quantification of pathological alterations in cardiac tissue. This can consequently also facilitate the optimization of therapeutic strategies by allowing the visualization of the response to therapy at structural level in preclinical studies^37^.

## Methods

### Animal model

For the pressure-overload model, eight weeks old C57Bl/6N mice were operated with transverse aortic constriction as described previously^7,38^. Sham animals underwent the same procedure except the banding of the aorta. Mice were euthanized 16 weeks post TAC surgery and whole hearts were excised. All animal in-vivo procedures were performed in compliance with the guidelines of the European Directive (2010/63/EU) and the German ethical laws and were approved by the administration of Lower Saxony, Germany (No. 33.8-42502-04-17/2585).

### Sample preparation

Freshly explanted hearts were weighed, briefly washed in phosphate buffer saline (PBS) and then fixed in 4% formaldehyde solution (FA) overnight at room temperature (RT). Hearts were then cut into transverse tissue sections of varying sizes such that adjacent tissue sections from the same heart sample were used for X-ray diffraction, SHG imaging and histology (Fig. 1A). Briefly, for sectioning, hearts were embedded in 5% agarose and were cut into 30 μm and 1 mm sections using a vibratome (VT1000 S; Leica Biosystems). The 30 µm thick tissue sections were used for X-ray diffraction and 2P-LSM and were stored in PBS containing 0.04% sodium azide until the day of the experiment. The 1 mm sections were embedded in paraffin for histology.

### Scanning X-ray diffraction

The spatially-resolved X-ray diffraction experiments were carried out on 30 µm cardiac tissue slices, which were mounted between two polypropylene foils as described before in Nicolas et al*.* 2017 at the microfocus endstation of the beamline ID13 at the European Synchrotron Radiation Facility (ESRF)^12^. The undulator gap was adjusted to generate an X-ray beam with a mean energy of E = 13.0 keV and bandwidth of ∆E = 10^–4^ E, as defined by a channel-cut Si(111) monochromator. The beam was focused by a combination of compound refractive lenses (CRL) inside a transfocator. The beam size in the focus was estimated from vertical and horizontal scans across the edges of two gold wires and was determined at 2.9 μm (horizontal) × 1.4 μm (vertical). A helium-filled tube was placed directly behind the focus of the beam to suppress air scattering along the beam path until approximately 10 cm behind the focus position where a lead wire with a diameter of 1 mm was used to block the primary beam. A calibrated diode was used to determine the total photon flux I_0_ = 1.57 × 10^12^ photons s^-1^. Given E, I_0_ and an exposure time of 10 ms, one can estimate the dose D at D = I_0_ τ E μ/(ρ ∆x ∆y) = 2.54 MGy, where μ/ρ = 3.1582 cm^2^ g^-1^ is the mass absorption coefficient estimated for skeletal muscle and ∆x x ∆y the focal size of the beam (FWHM).

### Immunofluorescence staining

Agarose embedded 30 μm vibratome sections were washed three times in TBS-T solution (20 mM Tris, 150 mM NaCl, 0.1% Tween 20). The tissue sections were incubated free-floating in 500 μL of DRAQ5 (Biostatus, 1:1000) for nuclear staining and were mounted on a glass slide using Immu-mount (Thermo Fisher Scientific).

### Second harmonic generation imaging

SHG imaging was performed for label-free imaging of myosin and collagen on 30 µm sections stained with the nuclear dye DRAQ5. Images were acquired with a two photon laser scanning microscopy (2P-LSM) setup (TriM Scope II, LaVision BioTec) equipped with a femtosecond-pulsed titanium-sapphire (Ti:Sa) laser (Chameleon Ultra II; Coherent). A Zeiss W Plan-Apochromat 20x (NA 1.0) water immersion objective was used for image acquisition. Multi-photon signals from tissue sections were detected in the backward and forward direction. For excitation of the SHG from the cardiac tissue, the Ti:Sa laser was set at 810 ± 5 nm and the nuclei stain DRAQ5 was excited at 720 ± 5 nm. The emitted light was split by a 495 nm and T560 nm long pass dichroic mirror (Semrock). The SHG signal was collected through a 405 ± 5 nm (FF01-405/10; Semrock) bandpass filter at photomultiplier (PMT) detectors (Hamamatsu) in the forward direction. The emitted fluorescent signal from nuclear stain was detected at a GaAsp PMT detector (Hamamatsu) in backward direction. All images were collected and processed with ImSpector (LaVision BioTec) and Fiji^39^. For the overview images an image size of 336 × 336 µm with 1024 × 1024 pixels and a pixel dwell time of 2.1 µs was used. Magnified images at the region of interest (ROI) were acquired using 100 × 100 µm image size, 916 × 916 pixels and 5.5 µs pixel dwell time.

### Histology and immunohistochemistry

The paraffin-embedded 1 mm cardiac tissue slices from regions adjacent to tissue sections used for X-ray diffraction and SHG imaging were cut into 2 µm sections (Fig. 1A). The sections were deparaffinized and dehydrated followed by haematoxylin & eosin (H&E) and Masson’s Trichrome staining (MTS) was performed as described before^40,41^.

For immunohistochemistry (IHC), deparaffinized sections were boiled for 20 min in Target Retrieval Solution (Dako) to perform the antibody staining with: anti α-SMA (clone 5694, abcam, 1:1000, overnight at 4 °C); anti CD68 (ab125212, abcam, 1:500, 1 h at 37 °C) and anti CD45 (clone 30-F11, 1:250, overnight at 4 °C) to identify the presence of activated fibroblasts, macrophages and leukocytes, respectively. After washing, the secondary antibody (anti-rabbit-horseradish-peroxidase, undiluted, Histofine) was added for 30 min at RT. Sections were counterstained with haematoxylin.

The images were acquired with an Axiovert 200 M inverted microscope (Carl Zeiss Microscopy GmbH). Image generation and processing were performed with the software AxioVision Rel.4.6 (https://carl-zeissaxiovisionrel.software.informer.com/4.6/) and Fiji^39^, respectively.

### Statistical analysis

Maps of structural parameters obtained from the X-ray diffraction data and presented in Fig. 2 were segmented semi-automatically using k-means clustering. A five-dimensional dataset consisting of five parameters used in least-square fitting of the one-dimensional structure factors was used for the analysis and three clusters were isolated. Numerically, the k-means implementation of the open source library VLFeat ^42^ was used for this task. Histograms of lattice spacing shown in Figure S5 were modeled using a pseudo-Voigt profile, while the mode of the distribution was obtained from a kernel density estimation using the function ksdensity available in Matlab 2018b (MATLAB, Mathworks Inc). A two-tailed paired Student’s t-test implemented in MATLAB with a p-value of 0.05 (*) as margin for statistical significance was used for the statistical analysis of the data.

### Ethical approval

Ethical permission for animal experiments was granted by the Nieders. Landesamt für Verbraucherschutz und Lebensmittelsicherheit (approval number 33.8-42502-04-17/2585).

## Supplementary information

Supplementary Information.

## Data Availability

Azimuthally averaged diffraction is available by the authors on demand.
