# Supplementary material for: X-ray diffraction and second harmonic imaging reveal new insights into structural alterations caused by pressure-overload in murine hearts
Source: Sci Rep. 2020 Nov 9;10:19317. doi: 10.1038/s41598-020-76163-6 (PMC7653033; doi:10.1038/s41598-020-76163-6)
Supplement: Supplementary file 1 — Supplementary Information. [file 41598_2020_76163_MOESM1_ESM.docx]

**X-ray diffraction and second harmonic imaging reveal new insights into structural alterations caused by pressure-overload in murine hearts**

Jan-David Nicolas^1,†^, Amara Khan^2,†^, Andrea Markus^2^, Belal A. Mohamed^3^, Karl Toischer^3^, Frauke Alves^2,4*^ and Tim Salditt^1,5*^

* Correspondence: tsaldit@gwdg.de and falves@gwdg.de

† shared first co-authorship

* shared last co-authorship

^1^Georg-August-Universität Göttingen, Institute for X-ray Physics, Friedrich-Hund-Platz 1, 37077 Göttingen, Germany.

^2^Max-Planck-Institute for Experimental Medicine, Translational Molecular Imaging, Hermann-Rein-Straße 3, 37075 Göttingen, Germany.

^3^University Medical Center Göttingen, Clinic for Cardiology and Pneumology, Robert-Koch-Straße 40, 37075 Göttingen, Germany and DZHK (German Centre for Cardiovascular Research), partner site Göttingen, 37075 Göttingen, Germany.

^4^University Medical Center Göttingen, Institute for Diagnostic and Interventional Radiology & Clinic for Hematology and Medical Oncology, Robert-Koch-Straße 40, 37075 Göttingen, Germany.

^5^University Medical Center Göttingen, Cluster of Excellence “Multiscale Bioimaging: from Molecular Machines to Networks of Excitable Cells”, Robert-Koch-Straße 40, 37075 Göttingen, Germany.

**Keywords:** Myocardial remodeling, label-free imaging, X-ray diffraction, muscle diffraction, two-photon laser scanning microscopy, second-harmonic generation

**Supplementary material**

**S1. Echocardiography and histological data for sham and TAC hearts**

Each TAC-operated mouse heart presented distinct severity of cardiac remodelling which is presented by the echocardiography functional analysis and histological data showing overview of cardiac morphology. All data for TAC hearts is presented in comparison to the sham hearts.

**Table S1:** Information on mice and hearts morphometry. Dates are given according to the international ISO 8601 standard, i.e. in the form yyyy-mm-dd.

| **ID** | **Sex** | **Date of birth** | **Opera-tion** | **Date of opera-tion** | **Date of sac-rifice** | **Body weight at harvesting (g)** | **Heart weight (g)** | **Long. Length of heart (mm)** | **Trans-verse width of heart(mm)** |
| --- | --- | --- | --- | --- | --- | --- | --- | --- | --- |
| Sham 1 | F | 2017-07-10 | Sham | 2017-09-04 | 2018-01-15 | 20.3 | 0.16 | 6 | 5 |
| Sham  2 | M | 2017-07-10 | Sham | 2017-09-18 | 2018-01-15 | 28.0 | 0.15 | 7 | 5 |
| Sham  3 | M | 2017-07-10 | Sham | 2017-09-18 | 2018-01-15 | 25.7 | 0.15 | 6 | 4 |
| Sham  4* |  | - |  | - | - | - | - | - | - |
| TAC  1 | F | 2017-07-10 | TAC | 2017-09-04 | 2018-01-15 | 19.3 | 0.33 | 10 | 8 |
| TAC  2 | M | 2017-07-10 | TAC | 2017-09-18 | 2018-01-15 | 26.7 | 0.36 | 11 | 9 |
| TAC  3 | M | 2017-07-10 | TAC | 2017-09-18 | 2018-01-15 | 30.0 | 0.32 | 10 | 8 |

* Data published in Nicolas *et al.,* 2017^12^.

**Table S2.** Echocardiographic parameters for sham and TAC mouse at 16 weeks after surgery

| **Mouse no.** | **HR**  (Bpm) | **LVIDd**  (mm) | **LVIDs**  (mm) | **PWThd**  (mm) | **PWThs**  (mm) | **AWThd** (mm) | **AWThs**  (mm) | **FS**  (%) | **EF**  (%) | **SV**  (µl) | **CO**  (mL/min) |
| --- | --- | --- | --- | --- | --- | --- | --- | --- | --- | --- | --- |
| Sham 1 | 437 | 4.0 | 2.9 | 0.6 | 0.9 | 0.8 | 1.1 | 27.5 | 52.4 | 41.8 | 18.3 |
| Sham 2 | 559 | 4.2 | 3.4 | 0.6 | 1.2 | 0.8 | 1.1 | 27.9 | 51.7 | 61.9 | 34.6 |
| Sham 3 | 470 | 4.0 | 2.5 | 0.8 | 1.5 | 0.9 | 1.0 | 38.7 | 66.3 | 56.9 | 26.8 |
| Mean  (n=3) | 489 | 4.08 | 2.92 | 0.69 | 1.17 | 0.84 | 1.09 | 31.4 | 56.82 | 53.5 | 26.54 |
| SEM (±) | 36.4 | 0.07 | 0.27 | 0.07 | 0.18 | 0.04 | 0.02 | 3.67 | 4.75 | 6.05 | 4.72 |
| TAC 1 | 445 | 4.4 | 3.8 | 1.0 | 1.1 | 1.1 | 1.3 | 11.7 | 31.2 | 33.8 | 15.0 |
| TAC 2 | 553 | 4.9 | 3.7 | 0.8 | 1.2 | 1.0 | 1.2 | 23.9 | 46.8 | 67.6 | 37.4 |
| TAC 3 | 468 | 4.7 | 4.0 | 1.0 | 1.1 | 1.1 | 1.5 | 15.2 | 39.3 | 52.0 | 24.3 |
| Mean  (n=3) | 489 | 4.6* | 3.9* | 0.9 | 1.1 | 1.1* | 1.4* | 16.9* | 39.1 | 51.1 | 25.6 |
| SEM (±) | 32.8 | 0.15 | 0.09 | 0.05 | 0.03 | 0.05 | 0.06 | 3.65 | 4.50 | 9.79 | 6.49 |

AWThd, left ventricle anterior wall thickness at diastole; AWThs, left ventricle anterior wall thickness at systole; bpm, beats per minute; Co, cardiac output; EF, ejection fraction; FS, fractional shortening; HR, heart rate; LVIDd, left ventricular internal diameter at diastole; LVIDs,  left ventricular internal diameter at systole; PWThd , left ventricle posterior wall thickness at diastole; PWThs, left ventricle posterior wall thickness at systole; SV, stroke volume; TAC, transverse aortic constriction. *P < 0.05 vs. sham; two-tailed unpaired Student&apos;s t-test. Data are expressed as mean ± SEM.


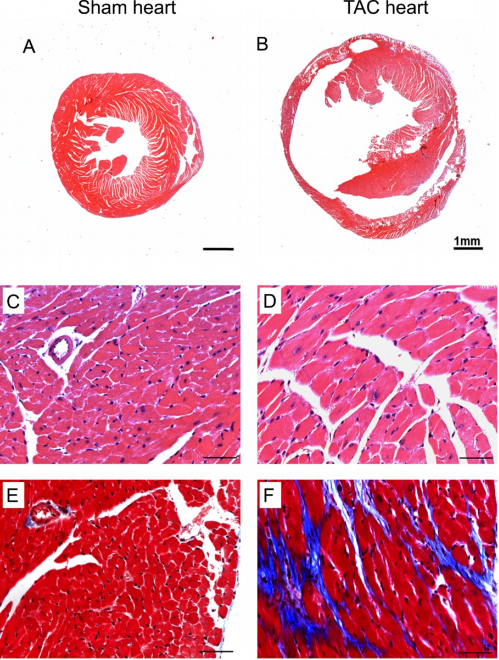


***Figure S1****: Histological representation of cardiac remodelling in transverse aortic constriction (TAC) heart. (A-B) Cardiac tissue sections stained with haematoxylin and eosin stain (H&E) shows increase in the overall size of a TAC heart in comparison to a sham heart. (C-D) Magnified images of H&E stained hearts showing the enlarged size of cardiomyocytes in the TAC heart as compared to the sham heart.  (E-F) Representative magnified images of Masson’s Trichrome stained (MTS) tissue from healthy sham heart with collagen (blue) deposition at the epicardium and in TAC heart MTS revealed development of interstitial fibrosis. Scale bar in A, B: 1 mm, scale bar in C-F: 50 µm.*


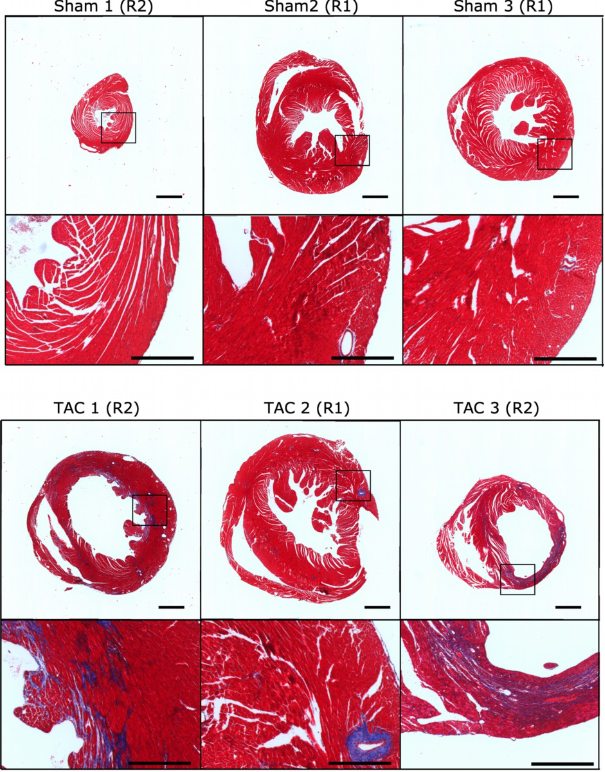


***Figure S2:****Overview and representative images at ROI of Masson Trichrome stained tissue sections from sham and TAC hearts. The muscle is stained pink while collagen is stained aniline blue.*

**S2. Quantification of lattice spacing difference in ring-like structural feature**

Three samples show significant alterations in lattice spacing within two regions of the myocardium. The region with lower lattice spacing appears in a ring-like shape and was found in samples from both sham and TAC and in region 1 as well as region 2. In all three samples, two smaller regions of interest (labeled A and B in Fig. S2) were identified by eye and the mean lattice spacings were obtained. It was found, that the lattice spacing differed by 1.7, 2.2 and 2.4 nm in region 1 of Sham 1, region 2 of TAC 1 and region 1 of TAC 1, respectively. The lattice spacing map relative to a reference value of 39.0 nm is shown for all three samples in Fig. S2.


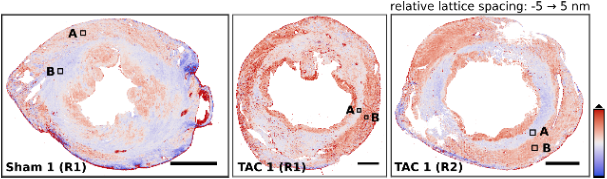
***Figure S3****: Three samples showed a reduction in lattice spacing within the myocardium with the shape of a ring. In region 1 of TAC 1 and region 2 of TAC 1, the boundary between these two regions appears sharp. The difference in lattice spacing was quantified by subtracting the mean lattice spacing obtained in region A from the mean lattice spacing of region B. Both regions are indicated by black boxes. Lattice spacing is given in nanometers, relative to a reference value of 39 nm.*

**S3. Quantification of anisotropy and lattice spacing in fibrotic lesion**

Regions of low intensity and low anisotropy pointed out in region 2 of sample TAC 1 and discussed in Fig. 3 of the main text appear to correlate well with the region of intense cardiac remodeling. Based on the two-dimensional (2D) histograms (or: 2D density of scan points) of anisotropy against lattice spacing and intensity, the difference in intensity and anisotropy between the fibrotic lesion and the bulk tissue can be quantified. Fig. S3A shows the 2D histogram of anisotropy against lattice spacing. Clearly, the bulk of the tissue has a lattice spacing of 39.5 nm and an anisotropy of 0.57, as identified by the maximum of the histogram which is highlighted by a red circle in Fig. S3. The distribution of lattice spacing also broadens with decreasing anisotropy. A decrease in anisotropy is, however, also linked to a decrease in intensity, as shown in Fig. S3B. Here, the distribution is tailed towards lower intensity with decreasing anisotropy. The maximum is now located at an anisotropy of 0.51 and an intensity of 0.27 counts/10 ms. To identify the region of cardiac remodeling, the lattice spacing serves as an accurate structural discriminator. Selecting only scan points with a lattice spacing greater than 45.0 nm yields the distribution shown in Fig. S3C, with a maximum at 0.12 and 0.11 counts/10 ms. Inspecting the logical map in (D) shows, that the affected region is indeed well localized by thresholding.


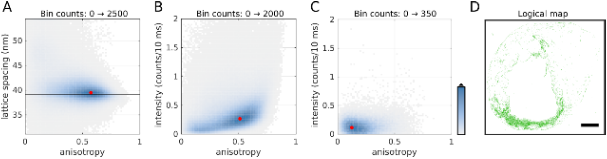
***Figure S4:****Estimation of intensity and anisotropy in the region of cardiac remodeling. (A) Density of scan points (excluding background) as a function of anisotropy and lattice spacing. The horizontal black line is located at a reference value of 39.0 nm obtained from six sham samples. A red dot marks the maximum of the density map. (B) Density of the same scan points shown in (A) plotted as a function of anisotropy and intensity, with a maximum at 0.51 and 0.27 counts/10 ms. (C) Selecting only scan points with a lattice spacing above 45 nm results in a distribution with a maximum at 0.12 in anisotropy and 0.11 counts/10 ms in diffraction intensity. Since the applied criterium coincides with the region of cardiac remodeling as seen in (D), it is the distribution of anisotropy and intensity within this region.*

**S4. Lattice spacing distributions and maps of structural parameters for all sample sections**

The entire X-ray dataset processed within the period of two allocated beamtimes consisted of 12 samples. All samples were processed in the same fashion and five structural parameters were extracted per sample. The lattice spacing, encoding the packing density of myofibrils, is shown in violin and box plots in Fig. S4A. It is evident, that some distributions, e.g. for TAC 1 (R1) are asymmetric. Due to this skewness, we have quantified the mode of all distributions instead of the mean. The average mode for all 6 Sham samples was found to be 39.0 ± 0.3 nm, and is indicated by a solid black line in Fig. S4A. To obtain the lattice spacing mode, the data was binned into a histogram with a bin width of 0.1 nm and the histogram was modeled using kernel density estimation (as implemented by the function *ksdensity* in MATLAB). The peak of the model function was chosen as the mode of the distribution. An initial attempt to quantify the distribution modeled the lattice spacing distribution as a sum of a Gaussian and Lorentz line shape with identical full width half maximum to include the longer tails of the distribution. The resulting pseudo-Voigt profile was fitted using a non-linear least-squares algorithm. In fact, in many samples the lattice spacing could be exactly modeled with this approach. It failed however for samples with significant skewness such as TAC 1 (R1) or Sham 1 (R1) or with more than one mode as in sample TAC 1 (R2) or, to a lesser extend, sample Sham 4. Data, kernel density estimate and model fit are shown in Fig. S4B. For completeness, the full dataset of all five structural parameters is on display in Fig. S5.


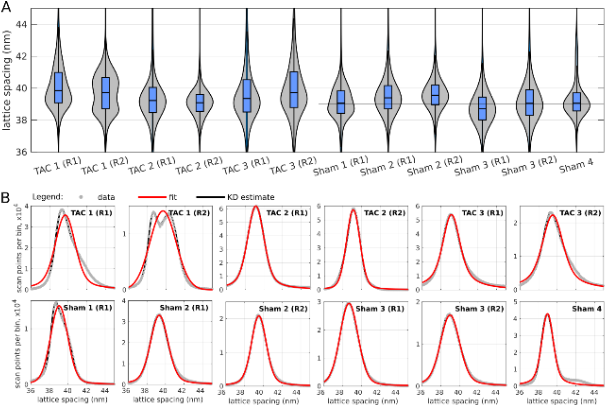
***Figure S5:****Analysis of the distribution of lattice spacings for all twelve imaged samples. (A) Violin and box plots of the lattice spacing distribution in 12 samples, out of which 6 samples were from three hearts of sham-operated mice, while 6 samples were from three murine hearts that showed hypertension and hypertrophy. Several samples showed an asymmetry in the lattice spacing distribution or even multimodality. The mean mode estimated from all Sham hearts was determined at 39.0 +- 0.3 nm. (B) The same distributions were modeled by a pseudo-Voigt profile using least-squares fitting and the mode was extracted from the maximum of the kernel density estimate.*


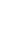

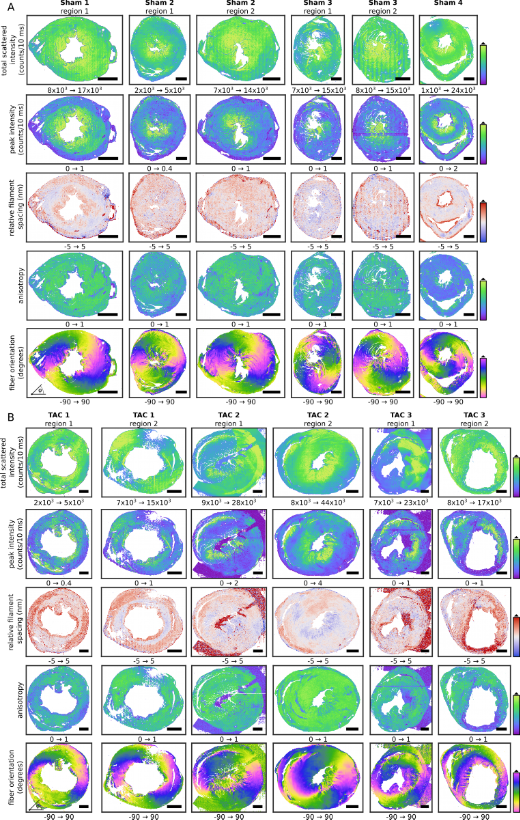


***Figure S6:*** *Maps of five structural parameters for all twelve imaged samples. (A) Sections from sham samples. (B) Sections from TAC samples.*

**S5. SHG micrographs for whole cardiac tissue sections**

2D SHG micrographs of whole cardiac tissue sections were stitched to present an overview of the myocardium structure in sham and TAC hearts.Myofibrils in sham hearts showed a symmetrical and regular arrangement while in TAC hearts a clear disruption of the myocardium was observed.


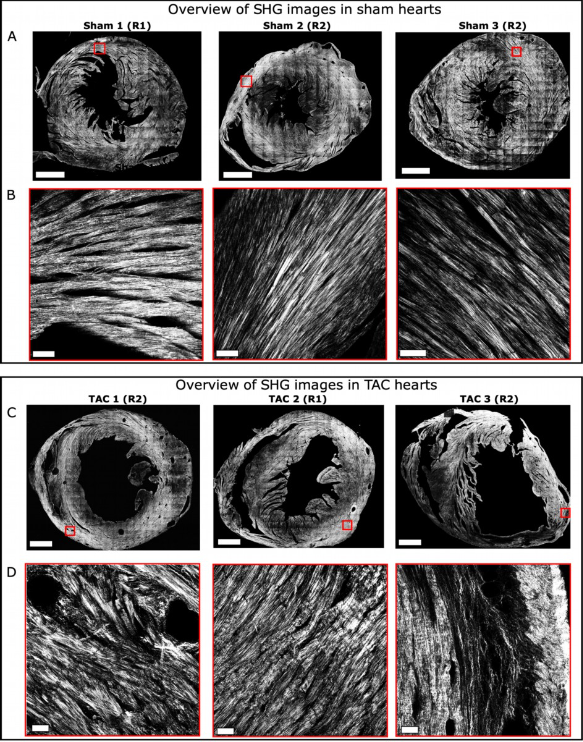


***Figure S7:****SHG imaging of sham and TAC hearts. Stitched 2D mosaic of whole cardiac tissue sections showing SHG in (A) sham and (C) TAC hearts. Representative images at ROI marked by a red box are shown for enhanced visualization of cardiac structures in (B) sham heart and (D) TAC heart. Scale bar in A and C: 1mm; scale bar in B and D: 100 µm*

**S6. Quantification of immune cell infiltration and alpha-SMA expression in TAC 3**

In order to quantify the cell infiltration in TAC 3 heart sample, the percentage increase in cell count was calculated at five ROI for both anti CD45 and anti CD68 stained tissue. An overall 78% increase in cell-infiltration was found in TAC 3 heart sample as compared to the sham hearts. The percentage area of positively labelled alpha-SMA was also measured for TAC 3 and sham hearts. The results showed a 65% increase in the expression of alpha-SMA in the myocardium of TAC 3 heart sample as compared to sham hearts.


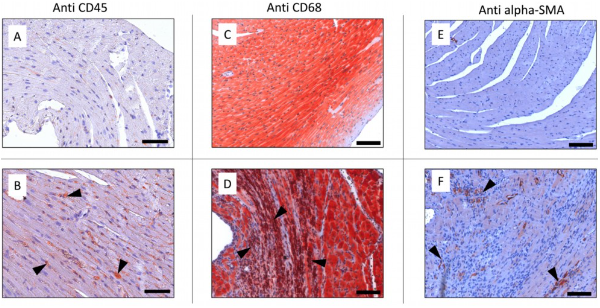


***Figure S8:****Representative images of IHC stained cardiac tissue sections a for sham hearts (top panel) and TAC hearts (bottom panel). (A-B) Image of anti-CD45 stained cardiac tissue revealing the infiltration of leucocytes in a representative TAC heart as compared to a sham heart. (C-D) Representative image of an anti-CD68 stained cardiac tissue shows an increased number of macrophages within the myocardium in a TAC heart as compared to sham heart. (E-F) Anti alpha-SMA staining shows cells positively labelled with alpha-smooth muscle actin in the myocardium of a TAC heart reflecting activated fibroblasts which are not present in the sham heart. The images represented are shown from sham 2 and 3, and TAC 3 heart samples. Scale bar: 100 µm.*

**S7. Quantification of perivascular fibrosis**

The quantification of perivascular fibrosis was performed on SHG and MTS images of sham and TAC heart using ImageJ software. Briefly, an ROI was selected around the blood vessels. A threshold was applied at the ROI to select the collagen emitted SHG and aniline blue stain for collagen in MTS images. The percentage area was then depicted which was used for statistical analysis. A two-tailed Student’s t-test was applied which indicated significant differences in collagen deposition between sham and TAC hearts for both SHG imaging (p=0.015) and MTS (p=0.0391).


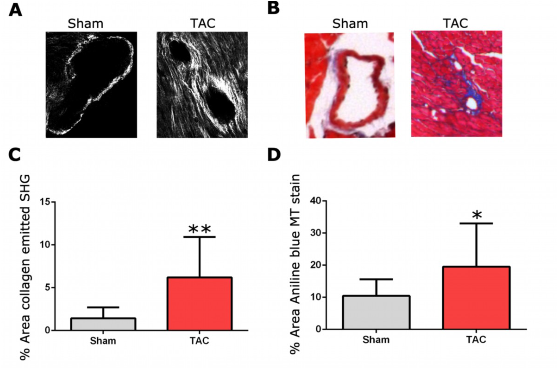


***Figure S9:*** *Quantification of perivascular fibrosis in sham and TAC hearts. A) Representative zoomed SHG images of coronary blood vessel in TAC and Sham heart. B) Representative zoomed images of MT stained blood vessel. C) Graph showing significantly higher percentage area of collagen emitted SHG in TAC heart as compared to the sham heart. D) Graph showing significantly higher percentage area for Aniline blue stain for collagen. The represented SHG and MTS images are from same sham and TAC sample. Data are the mean ± SE. *P<0.05; n = 3 per group.*

**S8A. 2D micrographs used for quantification of myofibril periodicity in sham hearts**

All images in section S8A and S8B were acquired with a two photon laser scanning microscopy (2P-LSM) setup (TriM Scope II, LaVision BioTec) equipped with a femtosecond-pulsed titanium-sapphire (Ti:Sa) laser (CRONUS laser; Coherent). A Zeiss W Plan-Apochromat 20x (NA 1.0) water immersion objective was used for image acquisition. All images were collected and processed with ImSpector (LaVision BioTec) and Fiji ^36^. Images at the region of interest (ROI) were acquired using 112 x 112 µm image size, 1024 x 1024 pixels and 2.25 µs pixel dwell time.


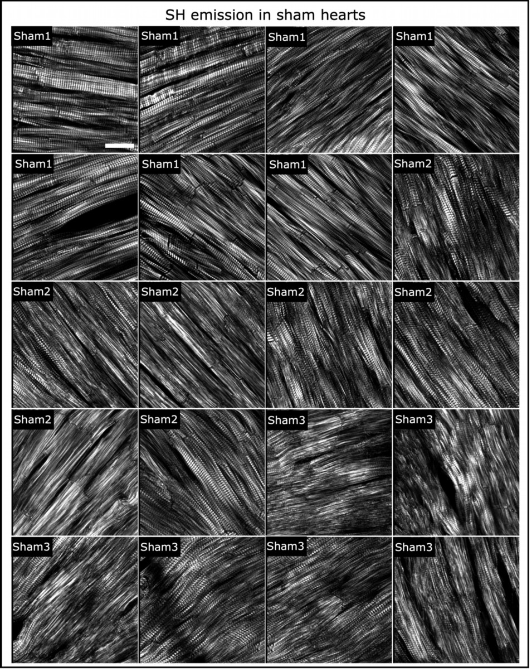


***Figure S10:****Twenty SHG micrographs showing the morphology of cardiac myofibrils in sham hearts (n = 3). All these images were used for quantitative and statistical assessment for determining the PSD indicator reflecting the orientation of strand, the striation periodicity in terms of the corresponding lattice peaks, as well as the degree of undulation in sham hearts. Scale bar: 25µm*

**S8B. 2D micrographs used for quantification of myofibril periodicity in TAC hearts**


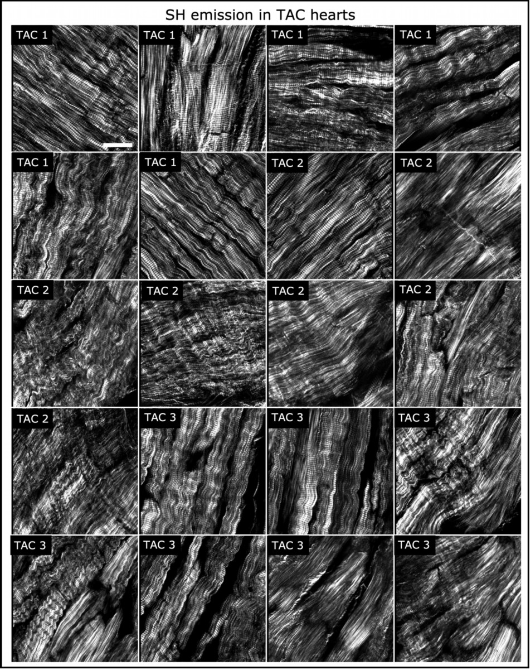


***Figure S11:****Twenty SHG micrographs showing the morphology of cardiac myofibrils in TAC hearts (n = 3). All these images were used for quantitative and statistical assessment for determining the PSD indicator reflecting the orientation of strand, the striation periodicity in terms of the corresponding lattice peaks, as well as the degree of undulation in TAC hearts. Scale bar: 25µm*
